# A cross-sectional study on the comparison of five ultrasound measurements to estimate central venous pressure in spontaneously breathing and ventilated patients

**DOI:** 10.1186/2197-425X-3-S1-A602

**Published:** 2015-10-01

**Authors:** N Parenti, ML Bacchi Reggiani, MG Vespignani, P Vernocchi, M Lanna, P Taffache, R Fiorito, M Metalli, G Rossi

**Affiliations:** Hospital Imola, Imola, Italy; University of Bologna, Bologna, Italy

## Introduction

The correlation of the inferior vena cava (IVC) diameter and the internal jugular vein (IJV) diameter with Central Venous Pressure (CVP) was already tested.

Few reports compared IVC and IJV ultrasound measures in the same population. No data there are on the correlation of the internal jugular vein diameter and CVP in ventilated patients.

## Objectives

To test and compare the correlation of CVP with IVC and IJV maximal diameters, collapsibility and distensibility indexes, IJV ratio in a population of ventilated and spontaneous breathing patients (SBP).

## Methods

This is a cross-sectional study conducted during 2015 in an Italian ICU. We included adult patients with a Central Venous catheter without cerebral ischemia or bradycardia.

Ultrasound images of IJV and IVC diameters at end-expiration (IJVDmax, IVCDmax) and at end-inspiration (IJVDmin, IVCDmin) were obtained with the patient supine using a 5-10 MHz and a 1-5 MHz probe (General Electric ).

At the same time: we performed a transthoracic echocardiography to evaluate the Left Ventricular Ejection Fraction and the Tricuspid annular plane systolic excursion (TAPSE) as index of Right Ventricular ejection fraction; we calculated the collapsibility index of IVC (IVCDmax - IVCDmin / IVCDmax X 100%) in SBP; the distensibility indexes (IVCDmax - IVCDmin / IVCDmin X 100%) in ventilated patients; the anteroposterior and transverse IJV diameters at end-expiration (AP-IJV, T-IJV) and the IJV ratio (AP-IJV divided by T-IJV); we collected clinical data (with intra-abdominal pressure and CVP). Analyses were performed using the Stata/SE Statistical Software 13.1. The Mann-Whitney test was used to compare variables between patient's groups. Correlations were calculated by means of Pearson or Spearman's rank correlation coefficients.

## Results

55 patients (12 ventilated and 43 spontaneous breathing) were included. The overall median age was 79 years (range 69-83), SAPS II was 36 (32-48), shock was main admission diagnosis. There were significant differences (p < 0,05) between patients with CVP ≤ 8 mmHg and those with CVP > 8 mmHg regarding IVCDmax, AP-IJV, IJV ratio (1,8 cm vs 2,2 cm; 6,8 mm vs 9,1 mm; 0,55 vs 0,67 respectively). There were no significant differences between ventilated and SBP patients regarding IVCDmax, AP-IJV, IJV ratio. We found a significant positive correlation between IVCDmax and IJV ratio and CVP in ventilated (r=0,61 with p=0,03 and r=0,6, p=0,04) as in SBP (IVCDmax vs CVP r=0,35, p=0,02; IJV ratio vs CVP r=0,35, p=0,03). AP-IJV was correlated to CVP in SBP (r=0,58, p=0,0001).

## Conclusions

If our results on the correlation of IVCDmax and IJV ratio with CVP in ventilated and spontaneous breathing patients will be confirmed in future studies, these measures could be use as alternative of CVP, in SBP could be used also AP-IJV. Limitation few ventilated patients.

